# Detection of postoperative granulation tissue with an ICG-enhanced integrated OI-/X-ray System

**DOI:** 10.1186/1479-5876-6-73

**Published:** 2008-11-27

**Authors:** Reinhard Meier, Sophie Boddington, Christian Krug, Frank L Acosta, Daniel Thullier, Tobias D Henning, Elizabeth J Sutton, Sidhartha Tavri, Jeffrey C Lotz, Heike E Daldrup-Link

**Affiliations:** 1 Department of Radiology, University of California, San Francisco, USA; 2 Department of Neurosurgery, University of California, San Francisco, USA; 3 Department of Orthopaedic Surgery, University of California, San Francisco, USA

## Abstract

**Background:**

The development of postoperative granulation tissue is one of the main postoperative risks after lumbar spine surgery. This granulation tissue may lead to persistent or new clinical symptoms or complicate a follow up surgery. A sensitive non-invasive imaging technique, that could diagnose this granulation tissue at the bedside, would help to develop appropriate treatments. Thus, the purpose of this study was to establish a fast and economic imaging tool for the diagnosis of granulation tissue after lumbar spine surgery, using a new integrated Optical Imaging (OI)/X-ray imaging system and the FDA-approved fluorescent contrast agent Indocyanine Green (ICG).

**Methods:**

12 male Sprague Dawley rats underwent intervertebral disk surgery. Imaging of the operated lumbar spine was done with the integrated OI/X-ray system at 7 and 14 days after surgery. 6 rats served as non-operated controls. OI/X-ray scans of all rats were acquired before and after intravenous injection of the FDA-approved fluorescent dye Indocyanine Green (ICG) at a dose of 1 mg/kg or 10 mg/kg. The fluorescence signal of the paravertebral soft tissues was compared between different groups of rats using Wilcoxon-tests. Lumbar spines and paravertebral soft tissues were further processed with histopathology.

**Results:**

In both dose groups, ICG provided a significant enhancement of soft tissue in the area of surgery, which corresponded with granulation tissue on histopathology. The peak and time interval of fluorescence enhancement was significantly higher using 10 mg/kg dose of ICG compared to the 1 mg/kg ICG dose. The levels of significance were p < 0.05. Fusion of OI data with X-rays allowed an accurate anatomical localization of the enhancing granulation tissue.

**Conclusion:**

ICG-enhanced OI is a suitable technique to diagnose granulation tissue after lumbar spine surgery. This new imaging technique may be clinically applicable for postoperative treatment monitoring. It could be also used to evaluate the effect of anti-inflammatory drugs and may even allow evaluations at the bedside with new hand-held OI scanners.

## Background

It is estimated that annually 8% of the working population in the US has lower-back related injuries [1]. A large proportion of these disabilities are related to vertebral disc herniations of the lumbar spine and can be treated by removing the protruded disk elements [2].

One of the associated risks of lumbar spine surgery is the development of postoperative granulation tissue. This granulation tissue may lead to postoperative complications such as, recurrent radicular pain, muscle weakness and paresthesia [3] and also contributes to further complications in the event of a follow up surgery [4-8].

Evaluation of disease progression and response to therapies is essential for treatment optimization and monitoring. Currently, the modalities used for imaging post-operative granulation tissue in patients includes, magnetic resonance (MR) imaging, computed tomography (CT) and SPECT/PET. However, each of these techniques is associated with shortcomings. Radiotracers can target granulation tissue with a high sensitivity [9,10], but SPECT and PET provide limited anatomical resolution and considerable radiation exposure. CT is readily accessible and offers excellent anatomical resolution, but is also associated with high radiation exposure [11]. MR has become the principal imaging technique for postoperative evaluations of the lumbar spine since it provides three-dimensional imaging data with excellent anatomical resolution and a high soft tissue contrast. However, MR is an expensive technique, which may be logistically complicated in post-surgical patients because it is not available at the bedside. In addition MR imaging may be confounded by potential artifacts due to surgical implants [12-20].

Optical imaging (OI) is a relatively new, inexpensive, fast, non-invasive and non-ionizing imaging technique based on the detection of fluorescence [21,22]. In order to enhance the contrast of OI, FDA-approved fluorescent dyes have been developed. Because these dyes accumulate in highly vascular areas visualization of granulation tissue with contrast enhanced OI can be done with high sensitivity.

A limited number of applications of OI for musculoskeletal disorders have been described so far, which is mainly due to the fact that this technique only allows for depiction of soft tissues and not the skeleton. To overcome these drawbacks, new integrated OI-/X-ray imaging systems have been developed that acquire and fuse optical images and X-rays. These fused OI-/X-ray images combine the high sensitivity of OI [23,24], with the direct depiction of the skeleton on X-rays. Our hypothesis was that these new integrated OI/X-ray systems provide a time- and cost-efficient approach for imaging granulation tissue after spine surgery.

Thus, the purpose of this study was to investigate the performance of an integrated OI-/X-ray imaging system for the diagnosis and localization of granulation tissue following lumbar spine surgery in a rat model. We determined the best timing and dose of an FDA-approved contrast agent that provided an optimal detection of postoperative granulation tissue on OI/X-ray images and then compared this data with histopathology. To the best of our knowledge, this is the first investigation of the performance of an integrated OI-/X-ray system for this application.

## Methods

### Animals and surgery

This study was approved by the committee on animal research at our institution. Eighteen male Sprague-Dawley rats (Charles River Laboratories, Wilmington, MA) aged 3 months and weighing 280–300 g were randomly divided into two groups of non-operated control animals (group A) and animals that underwent spine surgery (group B).

Prior to the surgical procedure each rat from group B received antibiotics (Trimethoprim-Sulfamethoxazole (Hi-Tech Pharmacal, Amityville, NY), 5 mg/kg, per os) and an intraperitoneal injection of buprenorphine (Reckitt Benckiser Pharmaceuticals Inc., Richmond, VA)(0.01–0.02 mg/kg). The animals were anesthetized with a single intraperitoneal injection of 35 mg/kg Sodium-Pentobarbital (Abbott Laboratories, Chicago, IL). After a vertical posterolateral skin incision and dissection through the left paravertebral muscles, the spine was exposed and a 20 gauge needle was inserted through the intervertebral disc at the level L2/3, keeping the annulus inside the cannula of the needle. The needle was advanced until it passed out of the posterior annulus as confirmed by fluoroscopy and then removed with the annulus inside.

At this point a second incision was made in the anterior portion of the upper tail in order to expose three tail intervertebral discs. A 16 gauge needle was passed through one of these discs, thereby collecting a portion of the nucleus pulposus. This material was reloaded into the above mentioned annulus-loaded 20 gauge needle. The loaded needle was then reinserted into the previously approached lumbar level (L2/3) and the needle contents (annulus and nucleus) were pushed with a stylus into the intervertebral disc of L2/3, thereby creating a disc protrusion and local granulation tissue.

After completion of this procedure, the abdominal wall and tail incisions were closed. Post-operative pain was controlled by intraperitoneal injection of buprenorphine every 8–12 hours for the first 48 hours. Medication with Trimethoprim-Sulfamethoxazole was continued for 72 hours post surgery (p.s.).

### Contrast medium

Indocyanine Green (ICG) is an FDA-approved approved, hydrophilic anionic near-infrared (NIR) dye with a molecular weight of 774.97 Da. The absorption and emission maximum wavelength of ICG are 805 and 830 nm respectively, which is within the NIR spectrum. ICG is rapidly cleared by the liver and bile fluid with a blood half-life of 3–4 minutes [25]. ICG shows a reversible plasma protein binding of up to 98% a few seconds after i.v. injection and a very low toxicity.

For this study, 20 mg of ICG (Fisher Scientific, Waltham, MA) was dissolved in 800 μl dimethyl sulfoxide (DMSO) (Fisher Scientific, Waltham, MA). This stock solution was diluted with saline to yield a 10 mg/ml or 1 mg/ml solution. In order to remove potential bacterial or dust contaminations, the solution was filtered through a 0.2 μm nylon filter (Alltech, Breda, Netherlands) directly before intravenous injection.

### In vivo imaging

**Figure 1:**
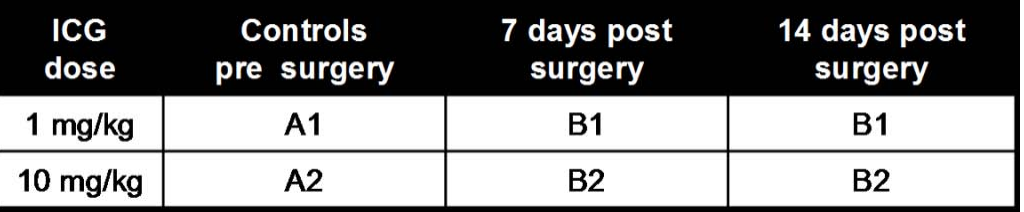
Overview of the different animal groups: the control group (A) and the experimental group (B), further divided into two dose groups, that received intravenous injections of 1 mg/kg (A1, B1) and 10 mg/kg (A2, B2) ICG.

All 18 rats were investigated with optical imaging (OI) and subsequent X-rays. The non-operated control group of six animals was divided further into two groups that received an intravenous injection of 1 mg/kg ICG (group A1, n = 3) or 10 mg/kg ICG (group A2, n = 3, Figure 1). Likewise, the animals of Group B, that had undergone lumbar surgery, were also divided into two groups that received either intravenous injections of 1 mg/kg (Group B1, n = 6) and 10 mg/kg ICG (Group B2, n = 6, Figure 1). The dose of 1 mg/kg was chosen as the typical dose currently applied for clinical applications [26,27] and the dose of 10 mg/kg was chosen as a dose previously used in rodents [28,29]. All animals in Group B underwent imaging studies at 7 days (n = 12) and 14 days (n = 12) after the spine surgery. Each imaging study of Group A and B consisted of the following protocol: (1) a pre-contrast OI scan, (2) ICG-injection, (3) OI scans from 1–25 min post injection (p.i.) and (4) X-rays at 30 minutes p.i.

For all OI scans, the animals were anaesthetized with 1.5 – 2.0% Isoflurane (Narkomed, Telford, PA) in oxygen. The rats were placed prone and lateral into the OI scanner (Imaging Station FX, Eastman Kodak Company, New Haven, CT). This OI system is equipped with a 150-W high-intensity halogen illuminator. For detection of ICG fluorescence, the excitation filter was set at 755 nm, the emission filter was set at 830 nm. Emitted light was collected using a thermoelectrically cooled CCD camera. The following imaging parameters were used for OI imaging: exposure time: 5 sec; F-stop: 0.0; FOV: 160 × 160 mm; focal plane: 5. Subsequent X-rays were obtained and digitized by the CCD camera. The following imaging parameters were used for X-ray acquisition: exposure time: 60 sec; F-stop: 3.7; FOV: 160 × 160 mm; focal plane: 5. OI scans and x-rays were merged with the Kodak Molecular Imaging Software 4.5 (Eastman Kodak Company, New Haven, CT).

In our optical imaging studies we encountered several difficulties with autofluorescence. Depending on the applied excitation and emission wavelength the skin and especially the hair of the animals were fluorescent and interfering with the signal of the deeper tissue e.g. the granulation tissue. When imaging at a lower wavelength we had to shave the animals in order to minimize the autofluorescence. However for this study we used a higher excitation (755 nm) and emission wavelength of (830 nm), and thus we could depict deeper tissue, such as granulation tissue with a low autofluorescence effect.

Following the last imaging session, the rats were sacrificed with an overdose of isoflurane and a bilateral thoracotomy. It is known that the signal intensity observed with fluorescence reflectance imaging varies with the depth of the target tissue. Therefore in order to study the biodistribution of ICG and to compare the signal intensities of the granulation tissue in vivo and ex vivo the lumbar spine (L3–L5) and organs (liver, kidney, spleen, bowl, lung, heart, bladder, urine and blood) were excised and imaged ex vivo with the OI/X-ray system. Then the specimens were processed for histopathology.

### Image analysis

Image analysis was performed by two observers in consensus. The optical images were evaluated qualitatively by assessing the presence or absence of visibly increased fluorescence in the region of surgery compared to normal contralateral muscle. An increased fluorescence of the left paravertebral soft tissues was interpreted as presence of postoperative granulation tissue. Quantitative analysis of OI scans was performed with the Kodak Molecular Imaging software 4.5. For each rat, the fluorescence signal intensity (SI) of the paravertebral granulation tissue and contralateral normal muscle was determined by operator defined regions of interest (ROI). This ROI was saved by the analysis software and applied to all other OI images of the same animal. For OI scans from different days, the same ROI was used, but manually repositioned by the operator in order to match the anatomical area of surgery. ΔSI was calculated by subtraction of SI of the postoperative granulation tissue from the SI of the normal muscle: ΔSI = SI _granulation tissue _- SI _normal muscle_. The relative fluorescence signal enhancement SI (%) of the left paravertebral granulation tissue was quantified as: ΔSI (%) = {(SI_post _- SI_pre_)/SI_pre_} × 100%.

### Histopathology

Lumbar spines and paravertebral soft tissues were harvested, placed in 10% non-buffered formalin and decalcified using Formical-4 (Decal Chemical Corp, Tallman, NY) for 2 days. Transverse sections were prepared through the levels of the previous surgery, including the spine and paravertebral tissues. The tissue was embedded in paraffin, sectioned in 5 μm thick slices, stained with H&E and Masson's Trichrome and evaluated using a Zeiss Axioskop 2 plus (Zeiss, Göttingen, Germany) at 1× and 40× magnifications. The presence, location and extent (diameter in cm) of the granulation tissue was determined for each animal and analyzed by a pathologist at our institution.

### Statistical analysis

All fluorescence data was presented as means and standard deviations of the means. Non-parametric Wilcoxon tests were utilized because it was not possible to determine whether the data were Gaussian distributed. A paired Wilcoxon test was used whenever there were repeated observations on the same animal. A standard Wilcoxon test was performed when comparing two different animal populations. Results were considered statistically significant if p < 0.05. All statistical computations were processed using SAS software (SAS Institute Inc., Cary, NC).

## Results

### In vivo studies

#### Pre-contrast versus post-contrast scans

**Figure 2:**
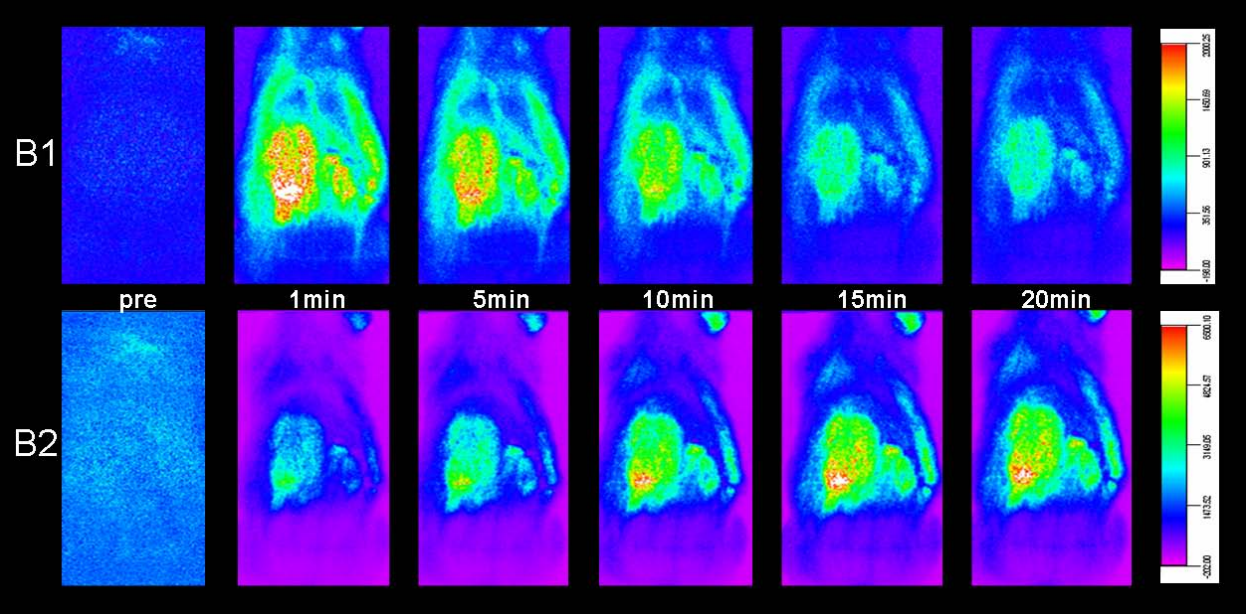
Dynamic optical images of the experimental animal group B, pre and at 1–20 min after injection of different doses of ICG: 1 mg/kg (B1) and 10 mg/kg (B2).

**Figure 3:**
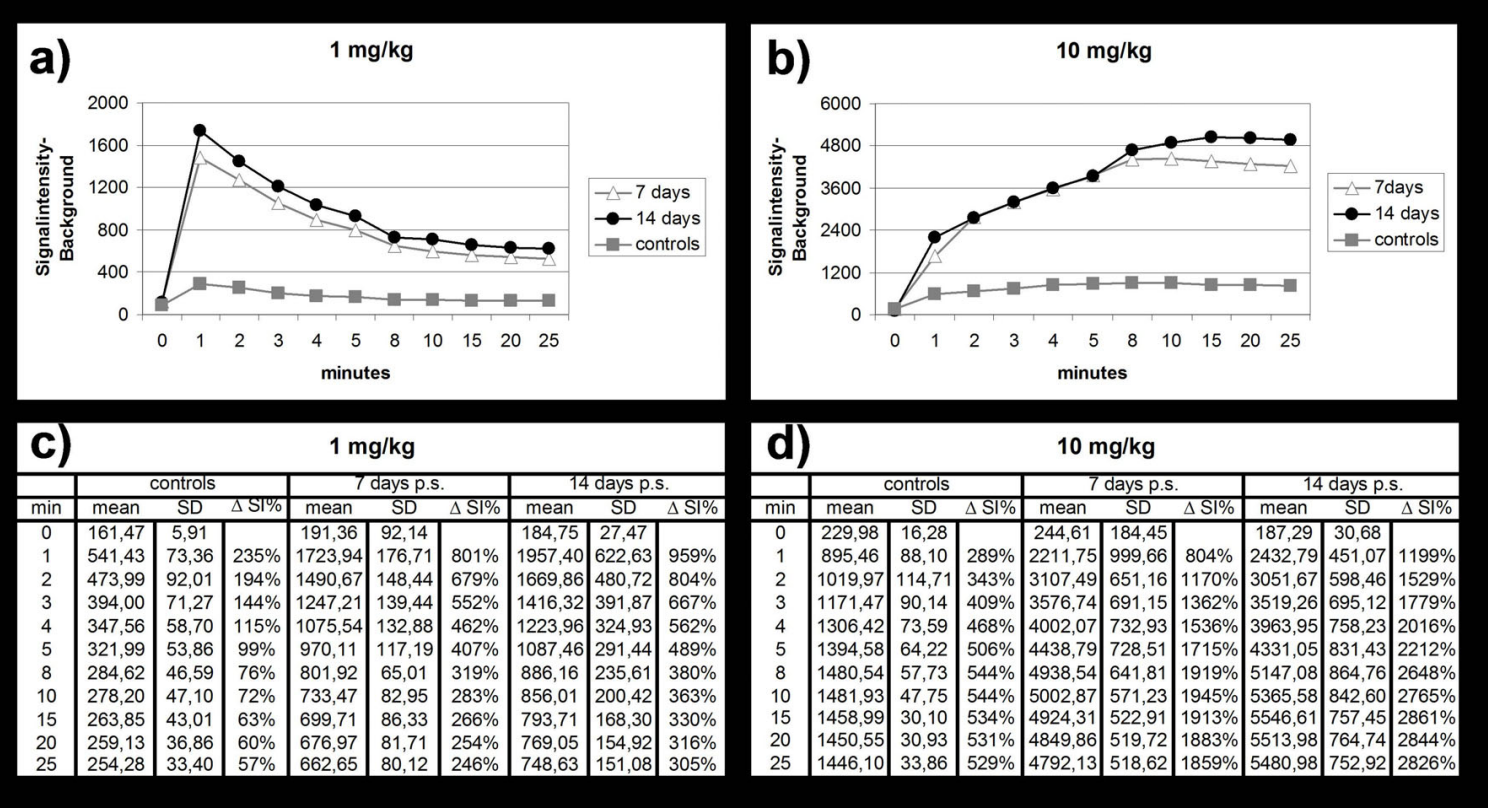
Mean fluorescence signal intensities subtracted from the background signal intensity (a, b) and corresponding quantitative data (c, d) of mean fluorescence signal intensities, standard deviation (SD) and the relative fluorescence signal enhancement (ΔSI%) of the paravertebral soft tissue in the area of previous surgery of the experimental group compared to the controls as measured before and continuously 1–25 min after injection of 1 mg/kg (a, c) and 10 mg/kg (b, d) ICG.

In all rats of the experimental group B, OI images showed a marked signal enhancement of paravertebral soft tissue at the area of surgery after intravenous injection of both administered ICG doses, 1 mg/kg and 10 mg/kg ICG (Figure 2, 3). Corresponding quantitative ΔSI data of the left paravertebral soft tissue were significantly higher on post-contrast images (B1: 1075 ± 207; B2: 4310 ± 695) compared to pre-contrast images (B1: 188 ± 60; B2: 216 ± 108) (p < 0.05). In rats of the control group A, OI images showed only a minimal and diffuse signal enhancement of paravertebral soft tissue after intravenous injection of both administered ICG doses. ΔSI data between pre- (A1: 161 ± 6; A2: 230 ± 16) and post-contrast (A1: 342 ± 56; A2: 1311 ± 63) images were significantly different (p < 0.05, Figure 3).

#### Comparisons between animals injected with different ICG doses

In animals of group B, the contrast agent kinetics of the left paravertebral soft tissues were different after injection of the two different ICG doses. Following injections of the low ICG dose (1 mg/kg), the area of surgery showed an early peak enhancement (7 days p.s.: 1723.9 units; 14 days p.s.: 1957.4 units) at 1 min after ICG bolus injection, followed by a rapid decline in fluorescence signal (Figure 2, 3). Following injections of the high ICG dose (10 mg/kg), the area of surgery showed a slowly progressing contrast agent accumulation with a delayed peak enhancement (7 days p.s. at 10 min p.i.: 5002.8 units; 14 days p.s. at 15 min p.i.: 5546.6 units), which was followed by a plateau phase (Figure 2, 3). Corresponding maximal quantitative ΔSI(%) data were significantly higher using 10 mg/kg (5547 ± 758) compared to 1 mg/kg ICG (1957 ± 623) (p < 0.05). In addition, the time interval of significant enhancement of granulation tissue was significantly longer after injection of 10 mg/kg compared to 1 mg/kg ICG (p < 0.05) (Figure 3).

#### Comparisons between group A and B

The fluorescence signal of the left paravertebral soft tissue in the area of surgery on post-contrast images was markedly higher in the animals in group B compared to animals in the control group A. Corresponding ΔSI% data of the left paravertebral area were significantly higher for animals from group B (B1: 1075 ± 207; B2: 4310 ± 695) compared to control animals in group A (A1: 342 ± 56; A2: 1311 ± 63) (p < 0.05).

### Fusion

**Figure 4:**
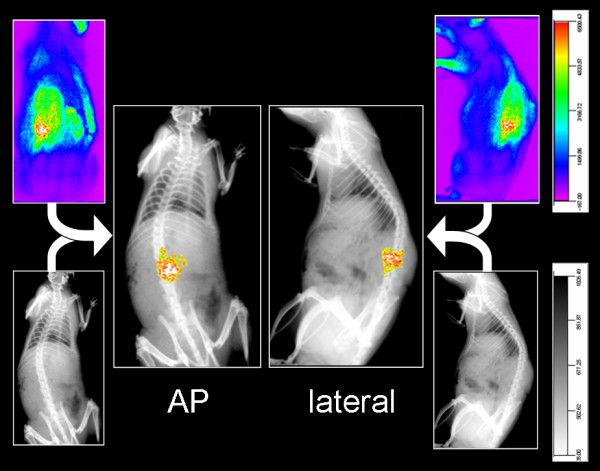
**Representative optical and X-ray images with subsequent fusion of a rat at 7 days post surgery, 10 min after injection of 10 mg/kg ICG, AP and lateral view.** In order to visualize the areas with the highest fluorescence after injection of the contrast agent fusion was performed by fusing all signal intensities above 6000 units on the OI image. Thus, the areas of highest fluorescence are visible on the fused image.

OI scans without X-rays did not allow an association of the area of fluorescence with the level of the lumbar spine. The Fusion of OI data with X-rays allowed an accurate anatomical localization of the enhancing granulation tissue (Figure 4). The enhancing left paravertebral soft tissue could be associated with adjacent lumbar vertebrae. This location corresponded to the area of surgery and the area of granulation tissue seen on histopathology.

### Ex vivo studies

**Figure 5:**
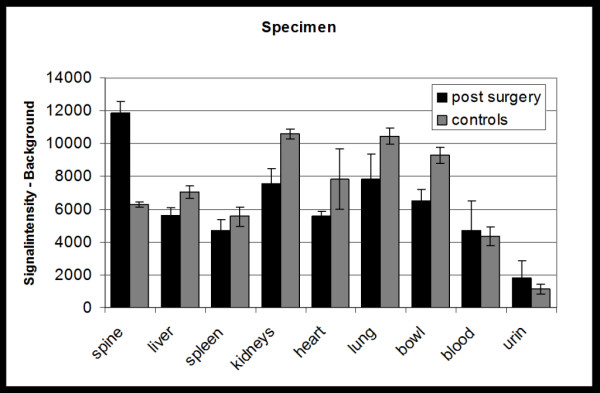
Mean signal intensities of excised organs of the experimental group B2 compared to the controls A2, measured ex vivo after previous injection of 10 mg/kg ICG (a). Representative optical images of excised organs of a rat of the experimental animal group (b).

Ex vivo OI scans of specimens (Figure 5) from rats of the experimental group B showed a higher enhancement of the spine at the location of surgery (11960 ± 695) compared to the enhancement of the corresponding area in the non-operated control group A (6398 ± 161) (p < 0.05). The enhancement of specimens of liver, kidneys, heart, lung, spleen, bowel, blood and urine were not significantly different in both animal groups (p > 0.05).

### Histology

**Figure 6:**
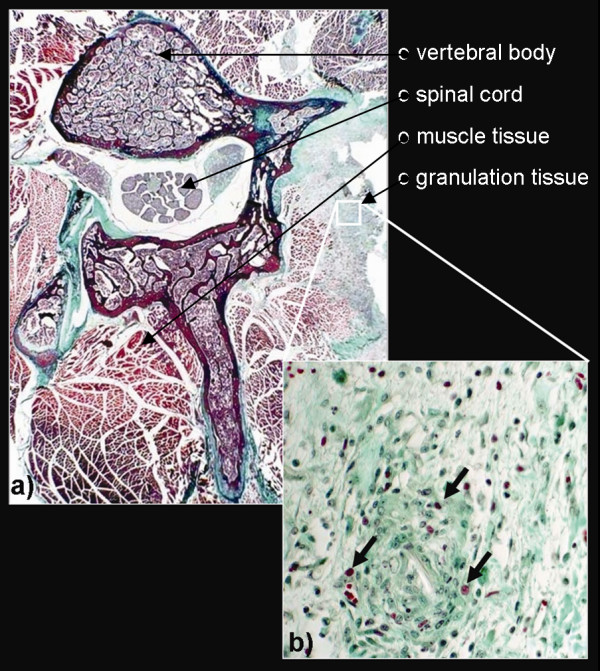
**Representative Mason's trichrome stains of the lumbar spine (L2/3) of the experimental animal group B show the development of granulation tissue at the left paravertebral soft tissue (a).** The magnification of the granulation tissue reveals numerous macrophages (arrows in b), being characteristic for the formation of granulation tissue.

Corresponding H&E and Mason's trichrome stains of the spine confirmed the presence of granulation tissue at the location of surgery (left paravertebral soft tissue adjacent to L2/3) in the experimental group B (Figure 6), while the control group A did not show any granulation tissue. The measurements of the granulation tissue resulted in a mean diameter of 3.1 mm (n = 12, standard deviation = 1.08).

## Discussion

This study showed that the investigated OI/X-ray system in conjunction with ICG-injection is a suitable technique to depict granulation tissue after spine surgery. Unique to this imaging system is its ability to acquire and fuse OI and X-ray images and thereby, facilitate an anatomical orientation with respect to the associated level of the lumbar spine. In addition, this investigation revealed certain advantages of using a high dose of 10 mg/kg of ICG, as opposed to a lower dose of 1 mg/kg. The dose of 10 mg/kg of ICG provided a stronger and prolonged enhancement of the granulation tissue thus allowing for longer observation times and improved detection of disease. Of note, the FDA approved ICG dose for clinical applications is 1 mg/kg. Although our data shows that this dose is sufficient to depict granulation tissue, future studies should evaluate if higher doses are also advantageous in the clinical setting.

The sensitivity of the OI/X-ray approach provides advantages over the current standard, MR imaging. T2-weighted MR images and gadolinium-DTPA-enhanced T1-weighted MR images reveal detailed information about the exact location and vascularization of granulation tissue as well as related displacement and thickening of nerve roots [30], but MR scans have a limited sensitivity. Peng et al. argued that standard clinical MR scans with 3–4 mm thick slices may not be able to detect small and poorly vascularized areas of granulation tissue [31]. Our study demonstrates that granulation tissue with an extent of 2–3 mm can be clearly depicted with OI. Furthermore, OI is easier to apply, faster (acquisition time is in the order of seconds) and is markedly less expensive compared to MR. In addition, new handheld OI scanners may allow investigators to perform studies at the bedside. Therefore, the high sensitivity of our OI technique provides an essential advantage for the detection of postoperative granulation tissue.

To the best of our knowledge, OI has not been used to image postoperative granulation tissue. However, other fluorescent dyes have been successfully employed for the detection of other chronic inflammations, such as arthritis [32,33]. ICG is superior to other fluorescent contrast agents for several reasons. ICG is FDA-approved for use in patients. It has been used to measure tissue blood volumes, cardiac output and hepatic function [34]. In addition, ICG has been applied for the detection of tumors [35-37], for angiography in ophthalmology [38] and for imaging of experimental arthritis [39]. ICG provides an excellent penetration depth of light in tissue because it displays strong absorption (~805 nm) and an intense emission spectra (~830 nm), which occur at wavelengths for which blood and other tissues are relatively transparent [40]. Finally, because of ICG's high affinity for blood proteins, it displays enhancement kinetics of a blood pool agent [41].

When applied in low concentrations, the majority of the agent stays in the intravascular compartment and, thus, leads to an early and short enhancement of the target tissue. Conversely, when applied in high concentrations, the biliary elimination of the agent is saturated, resulting in a prolonged circulation time and leaking across the hyperpermeable endothelium of the microvessels in the granulation tissue with every perfusion. This results in a slow accumulation of the agent in the interstitium of the granulation tissue, reflected by a slowly increasing and prolonged enhancement on OI. This prolonged enhancement of granulation tissue with the high ICG dose may be advantageous for potential future applications of handheld OI scanners, which are currently under development.

Our data showed that the integrated OI/X-ray system is particularly valuable for musculoskeletal and orthopedic applications. Potential drawbacks of the fusion technique could be misregistrations of the imaging data due to movement. Since our animals were anesthetized, we did not encounter any problems of this nature. However, potential clinical applications would have to provide an additional setup (e.g. holding devices) to avoid patient movement and consecutive misregistrations of imaging data. One limitation of our study is that we were not able to separate perivertebral and perineural granulation tissue because of the small anatomy of the rodent spine. Future clinical applications have to show, if the larger anatomy in patients will allow a separation of these two locations of granulation tissue.

With the number of clinical spine surgeries increasing every year, the management and treatment of postoperative granulation tissue is an increasing problem [2,4]. Treating this granulation tissue is of crucial importance in order to prevent complications in postoperative patients [42]. New anti-inflammatory therapeutics are currently being developed that aim to decrease the development and growth of granulation tissue and, thereby, decrease associated postoperative complications. The new OI-/X-ray technique, described in this study, will be applied as a non-invasive and cost-effective tool to directly and non-invasively monitor the efficacy of new anti-inflammatory drugs for the suppression of postoperative granulation tissue. In addition, the described OI technique is in principle ready to be applied in patients and could be used at the bedside once handheld OI scanners become available.

## Competing interests

The authors declare that they have no competing interests.

## Authors' contributions

JL and HD designed the study. FA and DT carried out the intervertebral disk surgeries. RM, CK and SB performed the optical imaging studies and acquired quantitative OI data. RM, TD, ES and ST performed the data analysis and histopathologic correlations. HD supervised all experiments. HD, RM and SB drafted and edited the manuscript. All authors read and approved the final manuscript.
